# Nano- and Microplastics in the Brain: An Emerging Threat to Neural Health

**DOI:** 10.3390/nano15171361

**Published:** 2025-09-04

**Authors:** Anna Baroni, Chantalle Moulton, Mario Cristina, Luigi Sansone, Manuel Belli, Ennio Tasciotti

**Affiliations:** 1 Human Longevity Program, IRCCS San Raffaele Roma, 00166 Rome, Italy; anna.baroni@sanraffaele.it (A.B.); chantalle.moulton@sanraffaele.it (C.M.); 2 Laboratory of Molecular, Cellular and Ultrastructural Pathology, IRCCS San Raffaele Roma, 00166 Rome, Italy; mario.cristina@sanraffaele.it (M.C.); luigi.sansone@sanraffaele.it (L.S.); manuel.belli@sanraffaele.it (M.B.); 3 Department of Human Sciences and Promotion of the Quality of Life, San Raffaele Roma Open University, 00166 Rome, Italy

**Keywords:** nanoplastics, microplastics, neurotoxicity, oxidative stress, neuroinflammation, neurodegeneration, mitochondrial dysfunction, rehabilitation

## Abstract

Nano- and microplastics (NMPs), with nanoplastics posing higher risks due to their smaller size and greater capacity for cellular and subcellular penetration, are being referred to as ubiquitous environmental neurotoxicants, due to their ability to pass through biological barriers, including the blood–brain barrier (BBB) and nasal olfactory epithelium, and to remain lodged in neural tissue. Upon uptake, such particles disturb neuronal homeostasis by multiple converging pathways, including oxidative stress, mitochondrial dysfunction, pathological protein aggregation, and chronic neuroinflammation, all closely involved with the molecular signatures of neurodegenerative disorders (Alzheimer’s, Parkinson’s, Amyotrophic Lateral Sclerosis—ALS). In addition to their neurotoxicity, recent findings suggest that NMPs could disturb synaptic communication and neuroplasticity, thereby compromising the brain’s capacity to recover from an injury, a trauma, or neurodegeneration, thus impacting the progression of the disease, our ability to treat it and eventually the efficacy of rehabilitation approaches. Despite these findings, our understanding remains hampered by analytical issues, the scarcity of standard detection methods, and a total lack of longitudinal studies in humans. This review combines multidisciplinary evidence on brain–plastic interactions and calls for accelerated advances in our ability to monitor bioaccumulation in humans, and to integrate neurotoxicology paradigms in the assessment of this underappreciated but growing threat to brain health.

## 1. Introduction

**Figure 1:**
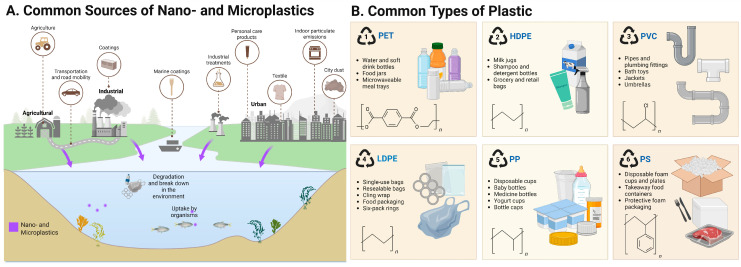
Figure outlining (**A**) the major types of plastic polymers and (**B**) common sources of nano- and microplastics (NMPs). PS—polystyrene; PE—polyethylene; PP—polypropylene; PVC—polyvinyl chloride; PET—polyethylene terephthalate; HDPE—high-density polyethylene; LDPE—ow-density polyethylene.

The last ten years have seen nano- and microplastics (NMPs) evolve very quickly from being an environmental problem to a significant public health issue [1]. Synthetic polymer particles, whether classified as microplastics (MPs, <5 mm) or nanoplastics (NPs, <1 μm), generally form from the breakdown of larger pieces of commercial plastic. Environmental levels of human-made NMPs have increased drastically over the last fifty years and, once released into the environment, NMPs are almost impossible to degrade, thus allowing them to penetrate air, water, soil, and food chains (Figure 1A) [2]. Increasing evidence points towards human exposure to NMPs largely through ingestion, dermal penetration and, more recently, through inhalation and even placental transfer [1,3,4]. Their appearance in human blood, placenta, lungs, and even more critically, brain tissues, establishes that these particles can penetrate protective physiological barriers and settle even in the most vulnerable and restricted tissues [5,6,7,8].

The central nervous system (CNS), whose integrity is guarded by the very selective blood–brain barrier (BBB), was long held to be impenetrable to the majority of environmental xenobiotics. Studies consistently identify two primary pathways for NMPs entry into the central nervous system: the systemic route, whereby particles enter the bloodstream and cross the BBB, and the nasal olfactory route, which allows direct translocation from the nasal epithelium into the brain via olfactory nerve fibres, and as a result bypasses the BBB entirely [9,10].

Recent reports describe that NMPs, especially particles <100 nm, cross the BBB by various mechanisms, such as transcytosis, endocytosis, and paracellular diffusion [11]. Alternative entry routes such as the nasal olfactory epithelium and the gut–brain axis offer other origin sites for NMP deposition in brain parenchyma [9]. Upon reaching the systemic circulation or neural interfaces, NMPs may be engulfed by brain-resident cells (e.g., microglia, astrocytes, neurons) and are trapped intracellularly since there are few exocytic pathways or degradation processes, ultimately resulting in accumulation in neural tissues [12]. The recent discovery of polystyrene (PS) NPs in human cerebrospinal fluid (CSF) and rodent brain tissues has steered the focus of NMP toxicology to neurotoxicity and neurodegeneration [13]. However, current understanding of long-term effects, biodistribution kinetics, and molecular mechanisms of NMP-induced neurotoxicity is limited, which underscores an urgent need for more comprehensive mechanistic studies and controlled models to ascertain their actual impact on brain health

Recent in vivo and in vitro studies have provided an initial mechanistic insight into the effects of NMPs on neural cells and the brain microenvironment. These studies show that NMP exposure induces oxidative stress through excessive reactive oxygen species (ROS) generation, impairs mitochondrial function, and disrupts autophagic pathways, ultimately leading to neuronal apoptosis [14]. These pathological mechanisms are consistent with those underlying neurodegenerative diseases such as Alzheimer’s disease (AD), Parkinson’s disease (PD), and amyotrophic lateral sclerosis (ALS) [15,16]. Apart from this, NMPs may also act as a platform for the misfolding and aggregation of amyloid-β, tau, and α-synuclein, which are marker proteins of AD and PD, respectively [17].

In addition to these disease-specific pathways, NMPs are increasingly implicated in more general dysregulation of synaptic transmission and neuroplasticity. NPs were reported by studies to affect dopaminergic, cholinergic, and glutamatergic neurotransmission and thereby disrupt cognitive and motor functions critical to everyday behaviour and potential for rehabilitation [18,19]. Microglial activation and neuroinflammation also have synergistic impacts on these effects, causing long-term structural and functional changes in the brain [12].

Despite this growing body of evidence, it remains difficult to fully clarify the impact of NMPs on brain health. Methods for detecting NMPs in tissues must still be developed, with Raman spectroscopy, FTIR, and mass spectrometry (MS) as the most established methods to date [20,21,22]. The lack of standardization, together with the heterogeneity in particle size, shape, polymer composition, and surface chemistry, complicates exposure assessment and risk characterization [23,24]. Epidemiologic evidence linking NMP exposure to neurological outcomes in humans is essentially lacking, highlighting the need for large-scale, longitudinal studies [25].

While recent reviews have summarized the environmental presence and general health effects of NMPs, it remains poorly understood how these exposures specifically drive neurotoxicity and, importantly, contribute to neurological pathologies, which this review aims to clarify [25,26]. Specifically, this review aims to provide a synthesis of current knowledge on brain-accumulated plastics. First, NMPs are categorized by size and polymer type, highlighting how these properties influence toxicity and bioavailability. Mechanistic links between NMP exposure and neurodegenerative processes are then examined, focusing on oxidative stress, mitochondrial dysfunction, neuroinflammation, protein misfolding, and synaptic loss. Finally, the potential impact of these disruptions on brain recovery and plasticity, particularly in the context of neurological rehabilitation, is discussed. By critically evaluating existing data and identifying research priorities across toxicology, neuroscience, and environmental health, this review shows that understanding NMP exposure is not only a scientific necessity but also a pressing public health need.

## 2. Types, Size, and Chemical Properties of Brain-Accumulated Plastics

### 2.1. Classification by Size

NMPs are usually separated based on size, which is a property that can affect possible uptake, downstream effects and toxicity [27,28]. MPs are usually considered particles between 1 µm and 5 mm, whereas NPs are below 1 µm, and for some, NPs are limited to <100 nm due to their high surface-area-to-volume ratio and unique physicochemical properties [5,29,30]. MPs of larger sizes are more likely to accumulate in peripheral organs, such as the gastrointestinal tract, liver, and spleen [31,32]. However, studies have shown that NPs smaller than 100 nm have the highest brain penetrance and preferential deposition in lipid-rich neural tissues [8,33]. Throughout this review, we use the term “NMPs” to broadly refer to plastic particles < 5 mm but with a specific focus on NPs < 100 nm due to their unique capacity to traverse biological barriers and access neural cells [5,29,30]. This size is commonly investigated since smaller NPs exhibit greater mobility, more pronounced protein corona formation, and increased potential for intracellular uptake and BBB traversal, compared with larger MPs. It should be mentioned that this difference is still to a certain extent artificial, as environmental samples and experimental systems often comprise a distribution of particle sizes that may have a range of biological behaviours.

Categorizing these particles based on size is critical since this can affect their ability to penetrate biological membranes and distribute throughout tissues. NPs, owing to their nanoscale dimensions, are more readily internalized by cells via endocytosis and can cross the BBB more easily, especially after the formation of a protein corona [34,35,36]. Kopatz and colleagues showed that NMPs can reach the brain within 2 h after oral administration, with the composition of the biomolecular corona playing a critical role in their passage through the BBB [11].

### 2.2. Types of Polymers and Additives

The chemical composition of NMPs found in biological tissue shows omnipresent polymer types, like PS, polyethylene (PE), polypropylene (PP), polyvinyl chloride (PVC), and polyethylene terephthalate (PET) (Figure 1B) [8,37]. Among these, PS NPs are employed most frequently in model systems due to their ease of access and their consistent morphology, and they are among the most frequently encountered in human and animal brain tissues. They are themselves not inert but, rather, can leach neurotoxic additives such as bisphenol A (BPA), phthalates, flame retardants, and UV stabilizers, all of which have independent neurotoxic properties [24,38,39]. BPA, for example, is a known endocrine disruptor with the capacity to bind the estrogen receptors in the brain and affect neurodevelopment [40]. Phthalates have also been shown to induce cognitive deficiency, oxidative stress, and behavioural disturbances in rodent models [41]. The combination of both polymers and leachable additives can yield synergistic toxicological profiles that are poorly represented by traditional toxicological assays.

### 2.3. Surface Functionalization and Bioreactivity: Contribution to Toxin Transport

The surface properties of NMPs, such as charge, hydrophobicity, and functional groups, control their biological reactivity and transport behaviour. Due to their high surface area, hydrophobicity, and capacity for surface functionalization, NMPs readily adsorb a wide range of environmental contaminants, including heavy metals (e.g., cadmium, lead, mercury), persistent organic pollutants (e.g., PCBs, PAHs), pesticides, and pathogenic microorganisms [42,43]. This adsorption not only increases the persistence of these toxicants in aquatic and terrestrial systems but also facilitates their transport and bioaccumulation across trophic levels [44,45]. As a result, NMPs can act as vectors of additive or even synergistic toxicity, where the combined effects of the plastic particle and its adsorbed contaminants exacerbate cellular stress responses, inflammatory signalling, and oxidative damage once internalized by organisms [42,45,46].

In biologic milieus, NMPs form a protein corona that can change their surface characteristics, providing a transient biological identity which allows them to engage with different types of cells affecting their recognition, uptake, and even facilitating transcytosis across the BBB [47,48,49]. Bioreactivity also depends on the local microenvironment; in some instances, inflamed tissues or acidic or oxidizing conditions have been shown to accelerate polymer degradation, producing reactive intermediates and promoting local toxicity [50]. These dynamic interactions among surface properties, environmental conditions, and host biology emphasize the complex risk profile of brain-collected plastics and serve as an imperative for nuanced characterization of their toxicological implications.

## 3. Presence of Microplastics in the Brain: Evidence and Detection

In recent years, numerous findings have provided compelling evidence that the brain is not exempt from plastic pollution and have emphasized the need for sensitive detection strategies and a mechanistic understanding of NMP neuroinvasion [51,52,53]. The detection of NMPs in human tissues has evolved from a speculative concern to established evidence, particularly regarding their presence in the brain.

### 3.1. Detection Techniques

**Figure 2:**
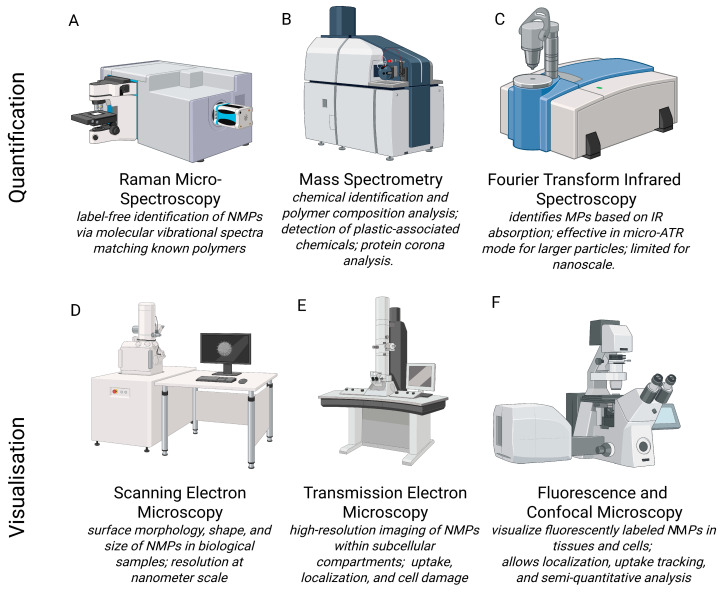
Illustration of instruments used for nano- and microplastic detection, quantification, or qualitative analysis. Quantification methods include (**A**) Raman Micro- Spectroscopy [55,56], (**B**) Mass Spectrometry [22], (**C**) Fourier Transform Infrared Spectroscopy [57,58] and visualisation methods include (**D**) Scanning Electron Microscopy [59,60,61], (**E**) Transmission Electron Microscopy [8,59,60,62] and (**F**) Flourescence and Confocal Microscopy [54].

Identifying NMPs in biological systems involves a variety of complementary techniques (Figure 2). Each method has its strengths in resolution, specificity, and chemical characterization. Fluorescence and confocal laser scanning microscopy are commonly used to visualize labelled particles within tissues and cells (Figure 2F). Roth et al. (2024) used this approach to track the uptake of 200 nm fluorescently labelled PS-NPs in human cell lines. This allowed for localization and semi-quantitative assessment [54]. Differently, other researchers have pointed out the increasing use of Raman micro-spectroscopy [55,56] and Fourier Transform Infrared Spectroscopy (FTIR) [57] for identifying NMPs without labels, especially in tissues like the placenta and lungs (Figure 2C). Raman techniques can detect particles down to 1 μm by matching molecular vibrational spectra with known polymer patterns (Figure 2A). FTIR, particularly in its micro-ATR mode, works well for characterizing slightly larger MPs, but it struggles with nanoscale particles due to diffraction limits [58].

Instead, electron microscopy, specifically scanning electron microscopy (SEM) and transmission electron microscopy (TEM), is an important tool for examining the shape, size, and structure of NMPs in biological samples (Figure 2D,E). Furthermore, these techniques provide insights into physiological and pathological phenomena that may go unnoticed with other experimental methods. Unlike optical methods, these techniques provide resolution down to the nanometre scale [59]. This allows the observation of the NMPs isolated or within cells, tissues, and extracellular matrices [60]. SEM is commonly used for imaging particle surfaces and is often combined with energy-dispersive X-ray spectroscopy (EDX) to confirm elemental makeup and differentiate plastic particles from biological or mineral debris [61].

TEM enables the detailed visualization of NMPs at a subcellular level. TEM imaging can be used to see how NMP particles are taken up by cells and where they are located within the cytoplasm. This method not only verifies particle uptake but also helps understand transport mechanisms and potential toxicity based on the localization of the particles in the cells. In addition, TEM allows the evaluation of stress responses within cells at subcellular levels, such as swelling of mitochondria, dilation of the endoplasmic reticulum, and lysosomal degradation. For example, TEM has been used to find and describe the presence of different-shaped plastic fragments in human placental tissues [62] and to evaluate the presence of NPs in postmortem human cortical tissue [8]. While SEM and TEM provide valuable information for detecting NMPs, there are some limitations. Sample preparation often requires dehydration and embedding, which can introduce artefacts or lead to particle loss. Additionally, these techniques do not have built-in chemical specificity unless used with methods like EDX or immunogold labelling. Therefore, it is best to use electron microscopy alongside vibrational spectroscopy (Raman, FTIR) or MS creating a multi-modal approach for accurately identifying and locating NMPs in biological systems.

### 3.2. Human and Animal Studies Showing Evidence of Nano- and Microplastic Accumulation

Experimental and clinical data suggest that NMPs can reach the brain through various routes. Inhaled PS-NPs may enter the brain via the nasal olfactory route, thereby completely bypassing the BBB [14]. Studies showed that intranasal administration or aerosol inhalation of PS-NPs, especially those below 100 nm in size, can lead to their direct transport into the brain [63,64]. Once in the brain, PS-NPs are taken up by neurons. This triggers inflammation, neurotoxicity, and behavioural changes in mice [64]. Smaller PS-NPs enter neurons even more efficiently and display a greater neurotoxic activity. Additionally, surface changes can affect brain uptake. Particles with amino groups (NH_2_) are taken up by brain tissue more easily than those with carboxyl groups (COOH). This difference likely comes from the positive surface charge of NH_2_-functionalized particles. This charge improves their interaction with the negatively charged cellular membranes and tight junctions of the BBB. These modifications may also affect protein corona formation, cellular signalling, and endocytic pathways. All these factors contribute to differences in toxicity and tissue distribution. These findings emphasize the importance of both particle size and surface properties in influencing the neurotoxic potential of NPs [14]. In an innovative study, Nihart and collaborators analyzed postmortem human samples and confirmed the presence of MPs, primarily PE, in the frontal cortex using pyrolysis–gas chromatography/MS, infrared spectroscopy, and electron microscopy. Their findings revealed nanoscale, shard-like plastic fragments distributed within the brain parenchyma, including in immune cells and cerebrovascular barriers [8]. In another study, the presence of various plastic polymers, such as PE, PP, and PVC, has been observed in human CSF, especially in patients with CNS infections [65]. At the same time, the buildup of PP, PE, and PVC in the CSF of the analyzed patients, whether they have CNS infections or not, did not significantly boost the production of important cytokines, specifically interleukin (IL)-6 and IL-8. However, the lack of a significant association between NMPs and cytokine levels may reflect the limited sample size, restricted disease spectrum included, limited cytokines analyzed, and cross-sectional design, which reduce the power to detect meaningful correlations. Despite this contrasting evidence, these findings suggest that some NMPs can enter the human central nervous system, especially when the BBB is weakened [65,66].

Since human data are limited, it is essential to examine findings from preclinical animal models. In vivo studies with rodents have shown that due to NMPs accumulating in brain tissue, they can induce functional impairments [67]. In a recent study, the long-term effects of lifelong low-dose exposure to PS-NPs in mice were evaluated. The exposure model mimicked real-world environmental levels. Mice were exposed from gestation through adulthood. The study showed that older mice accumulated more PS-NPs in the brain. These mice also had higher levels of neuroinflammation, oxidative stress, and changes in mitochondrial function. Interestingly, behavioural tests indicated increased anxiety-like behaviour and cognitive issues, especially in older mice [68]. This data emphasized that chronic exposure to low doses of NPs, even at levels lower than those used in toxicology studies, can cause subtle but progressive changes in brain function and behaviour throughout life [69].

### 3.3. Impact on Other Organs and Tissues

Contrary to the brain, larger MPs (often >100 μm) have been identified in liver, lungs, and other organs, indicating tissue-specific differences in plastic accumulation [11]. Furthermore, in terms of polymer type, PS-NPs are frequently reported in brain tissue, whereas PE and PP dominate in other organs [1]. The tissue-specific NMPs accumulation has raised numerous concerns about their toxicity mechanism.

In the respiratory system, MPs were found in all regions of the human lung. PP and PET were the most commonly detected types. Their distribution varied by location, suggesting that some areas are more vulnerable due to particle size and airflow patterns [70]. In the cardiovascular system, observational data from occupational exposure studies indicated that individuals exposed to plastics-related pollutants, such as PVC, may have a higher risk of developing cardiovascular disease compared to the general population [71,72]. Mechanistic findings in preclinical models suggest that the cardiovascular toxicity associated with NMPs may arise from both direct translocation of particles into the bloodstream and indirect pathways [73]. In a recent landmark study, Marfella and collaborators presented strong human data showing PE and PVC NPs in carotid artery plaques. In their prospective multicentre cohort of 304 patients undergoing carotid endarterectomy, MNPs were found in the atheromatous plaques of 150 individuals (58.4%), with polyethylene detected at an average of 21.7 µg/mg and polyvinyl chloride in 12.1% of cases at 5.2 µg/mg. By EM analysis, the research group observed irregular-edged plastic particles, mostly under 1 µm in size, embedded within macrophages and plaque debris, with some particles showing chlorine signatures consistent with PVC. Over a mean follow-up of 33.7 months, patients with detectable MNPs in their plaques had a 4.5-fold higher hazard of cardiovascular events (myocardial infarction, stroke, or death) compared to those without plastics in plaque (HR 4.53; 95% CI, 2.00–10.27; *p* < 0.001) [74]. The reproductive system also seems affected by NMP contamination. In 2023, Montano et al. found pigmented MPs in human semen, including particles of PS, PP, PE, and PET [75]. Additional evidence comes from another interesting study that discovered MPs in human placenta and breast milk, showing that these particles can cross placental barriers and be passed on after birth [4]. Their findings highlight the risk of early-life exposure to NMPs during important developmental stages. Other studies detected the presence of NMPs in liver, kidney, and spleen [76,77]. Together, these studies show that NMPs can spread to multiple organs, with growing evidence of localized inflammation and long-term health risks in different body systems.

Therefore, NMPs affect different organs in specific ways; the brain, in particular, has distinct vulnerabilities due to its unique immune status, the high lipid content of neurons, poor clearance from the brain, and the presence of strict barriers such as the BBB. In contrast, organs such as lungs, and the cardiovascular system, and reproductive tissues mainly show localized inflammation, oxidative stress, and reduced function after exposure to NMPs. The brain, however, seems to suffer more severely, showing, after long-term exposure, changes in synaptic structure, nerve cell death, and behavioural changes even at lower doses [1]. These variations indicate that even though NMPs affect many organ systems, the central nervous system might be especially susceptible to long-term and possibly irreversible damage. This highlights the importance of evaluating risk and understanding mechanisms specific to each organ.

## 4. Mechanistic Relationships Between Plastic-Induced Neurotoxicity and Neurodegenerative Disease

**Figure 3:**
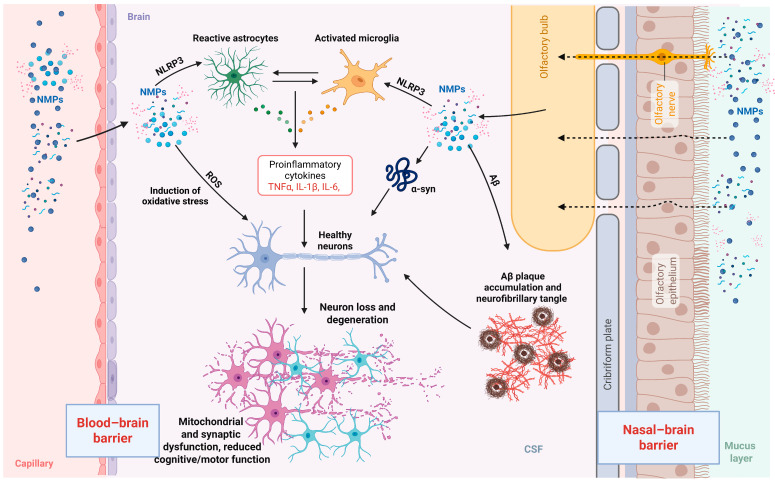
Mechanisms of nano- and microplastic (NMP) neurotoxicity. NMPs can enter the brain via two primary routes: translocation across the blood–brain barrier (BBB) and via the nasal–brain barrier through olfactory pathways. Once in the brain parenchyma, NMPs interact with resident glial cells, including astrocytes and microglia, leading to their activation and the release of pro-inflammatory cytokines. In parallel, NMPs induce oxidative stress and mitochondrial dysfunction. NMPs also induce oxidative stress, further contributing to a neuroinflammatory environment. In addition, NMPs may interact with pathological proteins, including alpha-synuclein and amyloid-beta, potentially exacerbating their aggregation and accumulation. These combined effects impair synaptic function and contribute to neuronal loss and degeneration, ultimately disrupting neuronal homeostasis and cognitive function. NMPs—nano- and microplastics; α-syn—alpha synuclein; PD—Parkinson’s disease; Aβ—amyloid-beta; TNFα—tumour necrosis factor alpha; IL—interleukin.

Due to their charge, size, and their properties of chemical-physical nature, NMPs can form such interactions with biomolecules that they can pass through the blood–brain barrier (Table 1) [10,13,78]. In turn, the crucial property of NPs is their large surface and hydrophobic nature, which makes them highly prone to absorb proteins [79]. Upon reaching the brain, NMPs may come in contact with neuronal proteins, for instance, β-amyloid and α-synuclein, that may thus be further aggregated [79,80]. Moreover, the connection of MNPs with the aberrant proteins can influence the protein degradation pathways and result in toxic protein accumulation. At the same time, the exposure to MNPs results in microglia activation, which consequently leads to the secretion of inflammatory cytokines and the process of chronic inflammation, along with an increase in the production of ROS. This reaction of events is the source of the increase in cellular damage, including mitochondrial dysfunction, DNA damage, and neuronal death. As a result, these processes play a very important role in assisting the neurodegenerative cascade, further exacerbating the progressive loss of neuronal function (Figure 3) [81].

### 4.1. Disruption of the Blood–Brain Barrier and Synergistic Neurotoxicity

The integrity of the BBB, a tightly selective barrier that protects the CNS from potentially toxic compounds, may be compromised under inflammatory, infectious, or metabolic conditions and, therefore, allow for penetration of exogenous substances such as NMPs. Several experimental studies have established that NPs of sizes less than 300 nm are endocytosed by BBB endothelial cells via several mechanisms like passive diffusion, clathrin- and caveolin-mediated endocytosis, and micropinocytosis [79].

Uptake via these mechanisms is dependent upon some particle attributes such as size, charge, and biomolecular corona structure. A key factor contributing to the complexity of NMP toxicity is the formation of the biomolecular corona, a layer composed of proteins, lipids, and other biomolecules that adsorb onto the NP surface upon entering complex biological environments [80]. This corona significantly alters the plastics’ biological identity and reactivity, thereby influencing their biodistribution, ability to traverse the BBB, and interactions with the cellular and molecular components of the CNS. Molecular dynamics simulations have demonstrated that the presence of cholesterol within the corona promotes the introduction of NPs into the lipid membranes of the BBB, thereby enhancing their neuroinvasive potential [11]. Protein–NP interactions are primarily mediated by van der Waals forces, hydrophobic interactions, and, in some cases, electrostatic bonds, all of which can disrupt the intramolecular interactions required to maintain proper protein folding and function [90]. This acquired molecular identity in the circulatory system is a critical determinant of the neuroinvasive and neurotoxic potential of NPs.

Furthermore, NMPs have been shown to trigger structural alterations in the tight junctions of brain endothelial cells, for example, reduced occludin expression and increased paracellular permeability [85].

Once the NPs have crossed the BBB, they can activate resident microglial cells by triggering an intense inflammatory response with elevated levels of tumour necrosis factor alpha (TNF-α), IL-1β, and ROS, which may lead to neuronal death. NMPs possess both direct and indirect neurotoxicity. For instance, medium preconditioned by NP-activated microglia was found to cause extensive damage to HT-22 neurons, indicating that there exists a secondary toxic effect mediated by the extracellular microenvironment [85]. Progressive disruption of the BBB can again reinforce NMP deposition within the brain. Clinical findings also revealed higher levels of PE, polypropylene PP, and PVC in CSF from infected or inflamed patients of the CNS. In both cases, BBB disruption was indicated by an increased albumin-CSF index [65]. Despite the controversial relationship of NMP appearance with inflammatory CSF markers, BBB impairment may facilitate their accumulation.

Overall, damage to the BBB acts as a permissive factor in NP invasion of the brain, while physicochemical properties and biomolecular corona of particles determine their mode and efficacy of entry. This results in cytotoxic synergy between increased BBB permeability and toxic molecular activities of NPs in the neural microenvironment. The BBB–NP axis is thus a critical but relatively unstudied axis for new environmental neurotoxicity research with significant consequences for the development of neurodegenerative and neuroinflammatory disorders.

### 4.2. Neuroinflammation and Microglial Activation

A central mechanism underlying the neurotoxicity of NMPs in mammals is the activation of microglial cells and the subsequent induction of neuroinflammation processes that are critically involved in the pathogenesis of neurodegenerative diseases such as AD, PD, and amyotrophic lateral sclerosis (ALS) [12,85]. In adult mouse models, microglia have been shown to internalize NPs, undergoing both morphological and transcriptional changes, which in turn stimulate pro-inflammatory responses in the brain [91].

Activated microglia release key pro-inflammatory cytokines, including IL-1β, TNF-α, and IL-6 [10,13,33,92]. Specifically, PS-NPs activate Toll-like receptor 4 (TLR4) in murine microglia, leading to recruitment of the adaptor protein MyD88 and subsequent activation of the IRAK/TRAF6 complex. This triggers phosphorylation of the IKK complex, which in turn promotes IκB degradation and allows nuclear factor kappa-light-chain-enhancer of activated B cells (NF-κB) translocation into the nucleus, where it drives transcription of pro-inflammatory cytokines such as TNF-α, IL-1β, and IL-6. Additionally, TLR4 activation contributes to mitochondrial ROS generation, further amplifying NF-κB signalling and sustaining a state of chronic neuroinflammation. As a result, NMPs lead to increased cytokine production, mitochondrial dysfunction, and further activation of NF-κB, a driver of chronic inflammation [91]. Additionally, NMPs have been shown to activate the NOD-, LRR-, and pyrin domain-containing protein 3 (NLRP3) inflammasome, an intracellular complex that processes IL-1β and IL-18, further amplifying the inflammatory cascade [69]. Broadening the picture, microglial cultures exposed to PS-NPs showed a marked reduction in spontaneous electrical activity in hippocampal neurons, indicating that neuroinflammation driven by microglial activation translates into measurable neurotoxicity [12]. Moreover, NMP exposure appears to exacerbate pre-existing neurological conditions. In a mouse model of global cerebral ischemia, administration of 0.5 µm microplastics led to increased microglial activation (as evidenced by Iba-1 and CD68 markers), elevated levels of IL-6 and TNF-α and heightened neuronal death [33].

Since microglial activation can have both protective and detrimental effects, contributing to both injury-induced repair and neuroinflammatory damage, NMPs may shift this balance towards degeneration, particularly following neurological insults such as stroke or traumatic brain injury (TBI) [93,94]. Rehabilitative medicine requires a favourable environment for motor and cognitive recovery; the imbalance caused by NMPs hinders the restoration of neuronal plasticity, axon regeneration, and synaptic remodelling, slowing down neurorehabilitation processes.

### 4.3. Oxidative Stress and Mitochondrial Dysfunction in Alzheimer’s and Parkinson’s Disease

ROS are byproducts of oxidative metabolism that play essential roles in various physiological processes, including cell signalling, immune defence, and apoptosis. However, when ROS levels exceed the cell’s antioxidant capacity, they induce oxidative stress, disrupting cellular homeostasis and contributing to the pathogenesis of numerous diseases.

NMPs cause cellular stress by disrupting mitochondrial function and altering energy metabolism, leading to excessive ROS production and structural mitochondrial damage [6,13,92]. ROS generation in response to plastic exposure has been observed across several cell types, including intestinal, hepatic, and neuronal cells, and is closely related to both dose and particle size [95]. For instance, exposure to NMPs in vitro results in mitochondrial impairment and the activation of inflammatory responses mediated by oxidative stress. The Nrf2 antioxidant pathway has been shown to be upregulated, likely in an attempt to counteract the damage, although it generally remains insufficient to restore mitochondrial integrity [96]. In vitro exposure of human cortical neurons to NMPs causes neurite shortening, degeneration, and a marked increase in mitochondrial ROS. Particles in the 20–100 nm range proved most neurotoxic, particularly when combined with pathogenic biofilms [97].

Mitochondria are central not only to energy production, but also to maintaining cellular homeostasis and neuronal survival. While ROS are naturally produced during oxidative phosphorylation, dysregulation of redox balance leads to cumulative damage. In neurodegenerative diseases, this redox imbalance is a core pathogenic mechanism [98].

In AD, ROS-induced mitochondrial dysfunction contributes to the accumulation of pathological proteins. β-amyloid aggregates interact directly with mitochondrial membranes, inhibit enzymes such as cytochrome c oxidase, increase ROS production, and perpetuate the oxidative stress cycle [99]. Furthermore, In PD, mitochondrial dysfunction is exacerbated by mutations in PINK1 and Parkin, genes crucial for mitophagy, the process of removing damaged mitochondria. Dysfunctional mitochondria continue to produce ROS, driving neuroinflammation, neuronal death, and irreversible brain damage. The situation is further worsened by mutations in mitochondrial DNA (mtDNA), which compromise mitochondrial receptor sites and further impair energy metabolism [100].

Together, these processes promote neuronal apoptosis and have been shown to exacerbate neurodegenerative pathology, potentially undermining the cellular recovery mechanisms that are critical for successful rehabilitation [10,101]. In the context of NMP exposure, these pathogenic processes are accelerated. Plastic particles that accumulate in brain tissue may exacerbate the same mitochondrial dysfunction, oxidative stress, and neurodegeneration observed in AD and PD.

### 4.4. Protein Misfolding and Aggregation in AD and PD

NPs pose a novel and growing challenge in the field of molecular toxicology due to their ability to interfere with the essential biological process of protein folding. The interaction with the surface of an NMP can disrupt the intramolecular interactions necessary to maintain protein’s correct three-dimensional structure as observed both in silico and in vitro [90]. Molecular simulations and experimental data have demonstrated that α-synuclein undergoes a conformational shift from an extended helical structure to a more compact aggregation-prone form when exposed to PE-NPs [102]. Similarly, β-amyloid shows an increased tendency to aggregate in the presence of PS or PE, with the nature of the interaction strongly influenced by the charge and composition of the NPs [80].

The protein misfolding induced by these interactions is typically irreversible and often leads to the formation of insoluble aggregates and fibrils that evade cellular degradation systems such as the ubiquitin–proteasome pathway and autophagy, leading to toxic accumulation in tissues. Animal studies have confirmed that NP exposure can activate the unfolded protein response, as evidenced by increased expression of markers such as ATF6α, HRD1, and CHIP in the lungs of mice exposed to PS-NPs [103]. This response indicates stress in the endoplasmic reticulum, the organelle responsible for protein folding, which attempts to restore homeostasis.

Overall, NPs function as mimetic molecular disruptors, interfering not only with protein structure but also with their function and turnover. Their capacity to induce protein misfolding and aggregation supports a potential causal link between plastic pollution and protein aggregation diseases such as AD, PD, and other systemic amyloidoses [79,104]. Prolonged exposure to environmental NMPs may be an emerging risk factor for neurodegenerative and systemic disorders, emphasizing the urgent need to investigate and mitigate the molecular-level impacts of plastic pollution.

### 4.5. Disruption of Neurotransmission and Synaptic Dysfunction

Experimental studies have demonstrated that NMPs can disrupt neurotransmitter synthesis and release, leading to impaired synaptic function and plasticity [10,13,101]. These effects are particularly relevant in the context of rehabilitation, as synaptic plasticity underlies the brain’s ability to reorganize and recover function after injury. Animal models exposed to MPs have shown decreased acetylcholinesterase activity, altered neuronal metabolism, and behavioural deficits, all of which could negatively impact recovery outcomes [13]. Astrocytes are glial cells of the nervous system, which actively participate in synaptic function by releasing neurotransmitters, regulating the ionic microenvironment and modulating synaptic plasticity. Chronic exposure to PS-NPs in an adult murine model led to anxiety- and depression-like behaviours associated with malfunctioning of the medial prefrontal cortex (mPFC). The study found lowered synaptic transmission and aberrant expression of the astrocyte marker GFAP, together with a considerable suppression of the glutamate transporter EAAT2, vital for synaptic glutamate clearance. Pharmacological activation of EAAT2 was able to restore both synaptic activity and behaviour, therefore underlining the crucial role astrocytes play in PS-NP-induced synaptic toxicity [105]. Neurotoxicity is also caused by secondary effects of MNPs; in zebrafish models, increased MNP-induced ROS production combined with weaker defences was linked to cognitive deficits, delayed neurobehavioral development, and motor deficits, and acetylcholinesterase strongly inhibits this behaviour, thus implicating oxidative damage in synaptic dysfunction [106,107].

Several mechanisms by means of which NPs interfere with neurotransmitter balance and synaptic function are mainly due to their effects on oxidative stress, neuroinflammation, receptor modulation, astrocyte dysfunction, and modified gene transcription. These consequences emerge as cognitive, motor, and affective impairments and may impact both adult organisms and their offspring, hence underlining the major environmental and public health hazards brought about by growing NP pollution.

## 5. Impact of Micro- and Nanoplastics on Neurological Rehabilitation and Recovery

**Figure 4:**
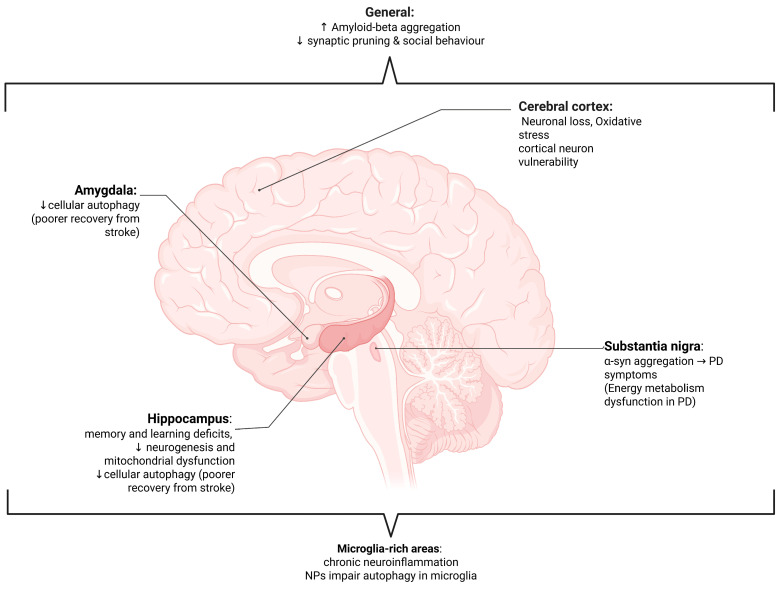
Diagram of the human brain showing the effects of nano- and microplastics (NMPs) on various areas of the brain and their functional implications. ↑, increase; ↓, decrease; α-syn—alpha synuclein; PD—Parkinson’s disease; NPs—nanoplastics. Effects are observed throughout the brain [107,108] and in microglial-rich areas [12,107,108], as well as in the cerebral cortex [97], amygdala [109], substantia nigra [110,111,112,113], and hippocampus [67,114,115,116].

NMPs have emerged as significant environmental neurotoxicants able to disrupt fundamental cellular and molecular processes within the nervous system (Table 2). These disruptions are particularly concerning in the context of neurological rehabilitation and recovery, where neuroplasticity, neurotransmission, and neuroimmune balance are essential for regaining lost function after injury or disease (Figure 4) [13,101].

**Table 2:** Summary of MPs/NPs effects on neurological disorders and brain function.

Condition	Plastic Type	Model/Sample	Mechanisms Involved	Observed Effects	Reference
Global cerebral ischemia	PS-NPs (0.5 μm) Dose: 50 mg/kg for one week	In vivo (Male Sprague Dawley rats)	Neuroinflammation, oxidative stress, microglia activation	Aggravate motor and cognitive impairment after ischemia ↑ Neuronal death, pro-inflammatory factors, ↓ Dendritic spine and synaptic proteins	Kim et al., 2025 [117]
Partial carotid ligation	NPs (50 nm) Dose: 25 mg/kg, 1 time/daily	In vivo, C57/BL6 mice	Neuroinflammation, inhibition of cellular autophagy, reduced synaptic plasticity and signalling	Worsened stroke recovery, toxic to hippocampus and amygdala ↑ Behavioural abnormalities (anxiety and depression) ↓ cellular autophagy (damage to hippocampus and amygdala)	Wang et al., 2025 [109]
Carotid artery plaque	Detection of NMPs (including PE and PVC)	In vivo, human (patients with carotid artery plaque)	Accumulation of NMPs	↑ risk of myocardial infarction, stroke, or death from any cause at 34 months of follow-up	Marfella et al., 2024 [74]
Alzheimer’s Disease	PS-NPs	In silico, in vitro, HMC3 (human microglial clone 3) cells	↑ Aβ aggregation, impaired microglial clearance, neuroinflammation, metabolic dysfunction	Accelerated disease progression ↑ Aβ-driven neuroimmune dysregulation	Wang et al., 2025 [108]
Parkinson’s Disease-like degeneration	PS-NPs (50 nm) Doses: 0.25, 2.5, 25, and 250 mg/kg daily (28 days)	In vivo, C57BL/6J mice	Mitochondrial and synaptic dysfunction, ↓ lysosomal degradation, energy metabolism disorders	PD-like symptoms, induced PD-like neurodegeneration by cell-specific pathways	Liang et al., 2022 [112]
Parkinson’s Disease	PS-NPs (amine surface modifications)	In silico, in vitro, primary neuron cultures In vivo, male wild-type outbred CD1 mice	Bind α-syn amphipathic and NAC domains, impaired lysosomal degradation, accelerate fibril-seeded α-synuclein pathology in neurons	↑ α-syn accumulation, α-syn spread throughout brain	Liu et al., 2023 [113]
Neurodegeneration	PS 2 µm MPs, 100 nm, and 20 nm NPs. Polyester MFs of ~10 µm (width) × 50 to 2000 µm (length) Dose: 0.68 mg/kg for 28 days (5 days on and 2 days of rest)	In vivo, C57BL/6J female mice In vitro, human bone marrow-derived neural precursor cells (NPCs)	Oxidative stress	Dose-, shape-, and size- dependent neurotoxicity and neurodegeneration. Cortical neuron vulnerability (nociceptive neurons are more resistant)	Vojnits et al., 2024 [97]
Cognitive dysfunction	5.0–5.9 µm MPs Dose: 0.01, 0.1 and 1 mg/day 30 days exposure	In vivo, male Kunming mice	↑ Escape latency (dose-dependent manner); SOD activity; MDA levels; ROS generation; AchE and ChAT activity ↓ Ratio of brain weight and body weight; GSH; acetylcholine	Oxidative stress Hippocampal cellular disorganization Cognitive deficits (learning and memory)	Wang et al., 2022 [118]
Cognitive dysfunction	25 nm PS-NPs Dose: 10, 25 and 50 mg/kg (6 months)	In vivo, male C57BL/6 mice	↑ Memory errors and incorrect movements; ROS generation Impairment in spatial learning and memory Synaptic and DNA damage	Cognitive dysfunction Neuroinflammation	Chu et al., 2022 [119]
Cognitive dysfunction	500 nm PS-NPs Dose: 0.1, 1 and 10 ppm (28 days)	In vivo, male Swiss albino mice	↓ Neuron and spine density at 1 ppm; number of Nissl bodies; BDNF expression Disruption of dendritic arborization Oxidative stress	Cognitive and neurological impairments Neurophysiological changes Impairment of synaptic activity and morphology	Suman et al., 2024 [120]
Motor dysfunction	100–500 nm PS-NPs and 1.0, 2.0 and 5.0 μm PS-MPs Dose: 1.0 mg L^−1^ (3 days)	In vivo, Caenorhabditis elegans	Neuronal damage, size-dependent toxicity	↓ Survival; size-dependent excitatory toxicity on locomotor behaviour; damage to cholinergic and GABAergic neurons	Lei et al., 2018 [27]
Cognitive dysfunction	PS-NH_2_ 50 nm NPs and 2 µm MPs Dose: 50 and 200 mg/kg (10 days)	In vivo, male C57BL/6 mice	↓ Cell proliferation Mitochondrial dysfunction Cell apoptosis	Memory impairment	Yang et al., 2023 [116]

↑, increase; ↓, decrease; NMPs—nano- and microplastics; PS—polystyrene; NPs—nanoplastics; MPs—microplastics; MFs—microfibers; PE—polyethylene; PVC—polyvinyl chloride; PS-NPs—polystyrene nanoplastics; PS-MPs—polystyrene microplastics; PS-NH_2_—amino-modified polystyrene; µm—micrometre; nm—nanometre; mg/kg—milligrams per kilogram; ppm—parts per million; Aβ—amyloid-beta; α-syn—alpha-synuclein; NAC—non-amyloid component; BDNF—brain-derived neurotrophic factor; AchE—acetylcholinesterase; ChAT—choline acetyltransferase; GSH—glutathione; SOD—superoxide dismutase; MDA—malondialdehyde; ROS—reactive oxygen species; NPCs—neural precursor cells; PD—Parkinson’s disease.

### 5.1. Evidence from Disease Models

#### 5.1.1. Stroke and Traumatic Brain Injury

After stroke and traumatic brain injury, neuroplasticity is fundamental for functional recovery. The overlap in mechanistic pathways, particularly microglial activation and neuroinflammation, suggests that chronic exposure to MPs and NPs could impair neuroplasticity and slow or limit recovery, although direct studies in this context are currently lacking [93,94]. NMPs may have negative effects on ischemic stroke in mice, potentially causing neurological deficits and larger infarct volumes [117]. Moreover, NPs can cause brain damage and accelerate atherosclerosis in mice with internal carotid artery occlusion, demonstrating the need for strategies to reduce NP intake in these patients [108]. Specifically, patients with carotid artery plaque containing NMPs have a higher risk of a composite of stroke, or death from any cause, at 34 months of follow-up [74].

#### 5.1.2. Neurodegenerative Diseases

NMPs can cause neurotoxicity and potentially contribute to neurodegenerative processes through their ability to cross the BBB and then interact with metalloenzymes [121,122]. These enzymes play vital roles in various brain processes, including neurotransmitter synthesis, energy metabolism, and protection against oxidative stress [123]. These enzymes are crucial for maintaining brain health and function, and their dysregulation can contribute to neurodegenerative diseases [123]. Minimal accumulation of NMPs can cause oxidative stress and neurodegeneration in human neurons, with cortical neurons found to be more susceptible to this damage in vitro [97].

In PD models, these plastics promote alpha-synuclein aggregation and dopaminergic neuron degeneration, effects mediated by both direct neuronal toxicity and gut–brain axis disruption [110,111]. Furthermore, PS-NPs can cause PD-like neurodegeneration in mice by causing energy metabolism disorders in the brain [112]. Additionally, NP pollution may promote PD-associated synuclein aggregation by interacting with protein fibrils and slowing down lysosomal degradation in neurons [113]. By directly promoting the progression of neurodegenerative diseases and inducing neural stress, these mechanisms could complicate rehabilitation and recovery in affected patients, contributing to poorer recovery and function.

#### 5.1.3. Effects on Cognition and Motor Function

Chronic exposure to MPs and NPs has been associated with cognitive impairment, memory deficits, and behavioural abnormalities in animal studies [13,64,92]. Interestingly, the post-mortem evaluation of the brains of human patients diagnosed with dementia showed that they had higher concentrations of NMPs, suggesting a possible link to neurodegenerative diseases [8].

Exposing mice to PS-MPs of different particle sizes (0.5, 4, and 10 μm) through drinking water for 180 days, resulted in a buildup of NMPs in the brain, along with damage to the BBB, inflammation in the hippocampus, increased levels of pro-apoptotic proteins, and a decrease in dendritic spine density. Behavioural tests indicated deficits in learning and memory, showing that plastics can harm the nervous system [67]. Additionally, exposure to PS-MPs of all sizes raised the levels of pro-apoptotic proteins in the hippocampus, suggesting that neurons become more susceptible to cell death. Reductions in dendritic spine density were also observed, which is critical for synaptic connectivity and plasticity. These changes are often linked to cognitive decline and intellectual disability [114]. It has also been shown that PS-NPs can cause cognitive dysfunction and microglial activation in the brain, potentially further contributing to cognitive impairment in neurodegenerative diseases [12]. Moreover, exposure to PS-MPs was shown to impair hippocampus-dependent learning and memory in mice [115].

Furthermore, exposure to NMPs can increase vulnerability to neuronal disorders and cause behavioural changes [6]. These particles have been shown to impair working and spatial memory and induce motor deficits, suggesting a direct threat to the efficacy of cognitive and motor rehabilitation in patients recovering from stroke, TBI, or neurodegenerative disorders [6,69,121,124]. Furthermore, it has been shown that cationic NPs impair hippocampal neurogenesis and memory retention by causing mitochondrial dysfunction in neural progenitor cells [116]. Adolescent exposure to NMPs leads to cognitive impairments in mice, with neuron loss and neurogenesis inhibition being more severe, and multi-omic alterations in the brain [109]. Overall, these findings are especially relevant because progressive neuronal loss is a key feature of many neurodegenerative diseases.

### 5.2. Plastics as an Emerging Barrier to Effective Neurorehabilitation

It is becoming clear that NMPs represent an underrecognized yet significant barrier to effective neurological rehabilitation. Their capacity to cross the BBB, disrupt neuroimmune balance, impair synaptic and mitochondrial function, and alter neurogenesis threatens the very biological processes that rehabilitation depends on, specifically cellular resilience, neuroplasticity, and cognitive–motor integration [6,10,13,64,92,101,125]. As these particles increasingly accumulate in human tissues due to pervasive environmental contamination, with particular accumulation in the brain at higher levels as compared with other tissues, individuals recovering from stroke, TBI, or neurodegenerative diseases may face added neurological burdens that are not addressed by current rehabilitative models [8,126]. The possibility that NMPs could blunt the efficacy of therapeutic interventions, delay recovery, or even worsen outcomes should prompt urgent investigation and public health awareness. In a world increasingly polluted by synthetic materials, recognizing plastics not only as ecological hazards but as potential disruptors of brain repair is essential for developing more resilient, personalized, and environmentally informed rehabilitation strategies.

## 6. Future Research Directions and Knowledge Gaps

Despite growing evidence of the pervasive presence of NMPs in the environment and their potential to affect human health, significant knowledge gaps remain, particularly regarding their neurotoxic effects. One of the foremost research priorities is the establishment of direct causal links between plastic exposure and neurodegenerative diseases [8,127]. While cross-sectional epidemiological studies have reported associations between microplastic exposure and increased prevalence of cognitive and functional impairments, these designs cannot confirm causality [10,128]. Longitudinal cohort studies are urgently needed to track individual exposure to NMPs over time and assess their potential role in the onset and progression of neurodegenerative disorders such as Alzheimer’s and Parkinson’s diseases [8,127]. Moreover, recent pioneering studies have detected MPs within human brain tissues, with notably higher concentrations found in individuals diagnosed with dementia [8]. These findings show the necessity of large-scale, multi-centre investigations employing standardized protocols to quantify microplastic burden in brain tissues and correlate these data with clinical and neuropathological outcomes. Additionally, a large body of evidence to date is derived from animal models, and their extrapolation to human health remains uncertain due to the absence of sufficient clinical correlation and the inherent limitations of translating preclinical findings into human risk assessment.

A critical barrier to advancing this field is the lack of standardized and sensitive methodologies for detecting and quantifying MNPs, especially at the nanoscale. Current analytical techniques, including micro-Raman spectroscopy, µ-FTIR, and pyrolysis gas chromatography–MS, exhibit substantial variability in sensitivity and reproducibility, particularly for particles smaller than 20 µm [129,130]. Inter-laboratory discrepancies further complicate the comparability of results across studies. To overcome these challenges, harmonization and standardization of sampling, sample preparation, and detection protocols are imperative [129,131]. Emerging technologies such as single-particle inductively coupled plasma MS and isotope labelling hold promise for improving the quantification of NPs but require rigorous validation before widespread adoption [129].

Another critical area for future research is the investigation of synergistic effects between microplastics and co-occurring environmental pollutants, such as heavy metals, pesticides, and organic contaminants [44,45]. MPs can adsorb and concentrate these toxicants due to their large surface area and physicochemical properties, effectively acting as vectors that facilitate pollutant transport and bioavailability [44,45]. Laboratory and environmental studies have demonstrated that interactions between MPs and heavy metals or pesticides can exacerbate toxicity, leading to increased bioaccumulation and adverse effects in aquatic and terrestrial organisms [10,44]. However, the implications of such combined exposures for human health, particularly regarding neurotoxicity, remain poorly understood and warrant comprehensive investigation.

## 7. Conclusions

In conclusion, NMPs represent an emerging and complex threat to human neurological health. NMPs can enter the brain, trigger inflammation, disrupt cellular health, exasperate neurological conditions, and impair cognitive and behavioural functions, which may hinder recovery from brain injuries or diseases. The extent to which they affect rehabilitation and long-term neurological outcomes in humans remains unclear. Analytical challenges, including the lack of standardized detection methods, and insufficient longitudinal human data currently limit the ability to fully assess risks. Furthermore, the potential for NMPs to interact synergistically with other environmental toxicants adds another layer of complexity that must be addressed. Given the urgency of this issue, there is a critical need for multidisciplinary collaboration among neuroscientists, toxicologists, environmental scientists, epidemiologists, policymakers, and industry stakeholders. Coordinated efforts in research, policy development, and public health interventions are essential to improve our understanding of NMP-induced neurotoxicity and to implement effective strategies for prevention and mitigation.

## Figures and Tables

**Table 1:** Table showing a mechanistic summary of the effects and presence of nano- and microplastics in the brain and brain cells in various models.

Model/Condition	Plastic Type	Particle Size	Exposure Route	Observed Effects	Reference
In vitro, mouse microglial cell line BV2	PS	2.5 µm	Direct exposure (1, 10, and 100 µg/mL)	↑ production and release of TNFα, IL-1β, and IL-6 ↑ pyroptosis-related proteins N-GSDMD and GSDMD	Wang et al., 2024 [82]
In vitro, mouse microglial cell line BV2	PS	480–30.3 nm	Direct exposure (25, 50, and 100 µg/mL)	↓ cell viability at 50 and 100 µg/mL ↑ NO and pro-inflammatory cytokines ↑ HRAS, Iba-1, NF-kB p65, and p-PERK levels	Li et al., 2024 [83]
In vitro, mouse microglial cell line BV2	PS	44 nm	Direct exposure (25, 50, and 100 µg/mL)	↓ cell viability ↑ ROS generation ↑ GPX4, XCT, ACSL4 ↑ NLRP3 and IL-1β in a concentration- and time-dependent manner ↑ ROS and MDA levels ↓ GSH and SOD in a concentration- and time-dependent manner Modification of ferritin transport proteins ROS levels reverted by NAC pre-treatment	Sun et al., 2023 [84]
In vitro, human cerebral microvascular endothelial cells (hCMEC/D3), murine microglia BV2 cells	PS	50 nm	Direct exposure (25, 50, and 100 µg/mL)	hCMEC/D3: NF-kB activation, ↑ TNFα levels, disruption tight junction ↓ TEER and expression of occluding ↑ ROS in a concentration- and time-dependent manner BV2 cells: ↑ TNFα and IL-1β; ↑ ROS generation	Shan et al., 2022 [85]
In vitro, hiPSC-derived cortical spheroids	PS-MPs	1–10 µm	Direct exposure (5, 50, and 100 µg/mL)	↑ expression of Ki67, MKI67, ATF4, Nestin, PAX6, HOXB4, and SOD2 ↓ cell viability; ↓ expression of TUBB3-TBR1/TBR2	Hua et al., 2022 [86]
In vitro, human neuronal cell line SH-SY5Y	PS	70–150 nm	Direct exposure	↑ Neurotoxicity of Aβ proteins, promoting the formation of pathogenic oligomers, oxidative damage, and neurological deficits	Gou et al., 2024 [17]
In vivo, male C57BL/6 mice In vitro, human neuronal cell line SH-SY5Y	PS	60–65 nm	In vivo: oral gavage In vitro: direct exposure in culture	In mice: cognitive deficits, neuronal loss, Nissl bodies in the prefrontal cortex ↓ GSH, SOD levels ↑ ERK/MAPK pathway, aggregation of lipolyzed proteins ↑ markers of cuproptosis (FDX1, LIAS, HSP70). In SH-SY5Y: ↓ cell viability, ↑ intracellular Cu^+^, ↑ FDX1, LIAS, HSP70	Chen et al., 2025 [41]
In vivo, 20 post-mortem human lung tissue samples	PP, 35.1% PE, 24.3% PVC, polystyrene polyurethane polyamide (<5%).	5.5–26.4 µm	Inhalation via the olfactory (nasal) route	Plasticity detected in 8/15 subjects; suggests transit via the olfactory nerve	Amato-Lourenço et al., 2024 [9]
In vivo, post-mortem human brain samples—frontal cortex	PE~75%, PP, PVC, SBR	<200 nm	Environmental exposure (inhalation + environmental ingestion), unspecified	Brain accumulation higher than in liver/kidney; correlation with dementia; particles in vessels and microglia	Nihart et al., 2025 [8]
In vivo, male C57BL/6 mice In vitro, HMC-3 cell line (human microglia)	PS	0.2 µm, 2 µm, 10 µm	In vivo: oral (gavage) In vitro: direct exposure in culture	↑ immune activation, apoptosis of microglia, inducing neuroinflammation and loss of support cells	Kwon et al., 2022 [87]
In vivo, male C57BL/6J mice In vitro, primary cultures of microglia, astrocytes, and hippocampal neurons	PS	30–50 nm	In vivo: oral (gavage) In vitro: direct exposure in culture	↑ microglial activation and inflammatory response ↓ neuronal activity Contribution to cognitive deficits	Paing et al., 2024 [12]
In vivo, male C57BL/6 mice In vitro, MN9D dopamine neuron line	PS	45–68 nm	In vivo: oral (gavage) In vitro: direct exposure in culture	↑ neuronal pyroptosis, blocking autophagic degradation through the TSC2-mTOR-TFEB pathway Progression of Parkinson’s disease	Liang et al., 2025 [19]
In vivo, male C57BL/6 mice	PS	80 nm	In vivo: oral (gavage)	Altered expression of neurotransmitter levels (5-HT, GABA) and nervous system proteins (AChE, BDNF, SYN, CREB) ↓ learning and memory ability ↑ Camk2g and Adcyap1 mRNA, ↓ Per1 mRNA in mouse hippocampus	Kang et al., 2023 [88]
In vivo, male C57BL/6 mice	APS (amino-modified PS)	100 nm	In vivo: oral (gavage)	↑ escape latency ↓ spatial learning and cognitive flexibility Memory and cognitive impairments Upregulation of AD mRNA markers ↑ Bax levels, ↓ Bcl-2 and NeuN levels ↑ iNOS, nNOS, Ac-Tau expression; ↓ Sirt1 expression	Bai et al., 2024 [89]

↑, increase; ↓, decrease; NMPs—nano- and microplastics; NPs—nanoplastics; MPs—microplastics; PS—polystyrene; PE—polyethylene; PP—polypropylene; PVC—polyvinyl chloride; SBR—styrene–butadiene rubber; APS—amino-modified polystyrene; µm—micrometre; nm—nanometre; NO—nitric oxide; ROS—reactive oxygen species; MDA—malondialdehyde; GSH—glutathione; SOD—superoxide dismutase; GPX4—glutathione peroxidase 4; XCT—cystine/glutamate antiporter; ACSL4—acyl-CoA synthetase long-chain family member 4; NLRP3—NACHT, LRR, and PYD domains-containing protein 3; GSDMD—gasdermin D; N-GSDMD—N-terminal gasdermin D; HRAS—Harvey rat sarcoma viral oncogene homologue; NF-κB—nuclear factor kappa-light-chain-enhancer of activated B cells; p-PERK—phosphorylated protein kinase R-like endoplasmic reticulum kinase; TEER—transendothelial electrical resistance; Aβ—amyloid-beta; FDX1—ferredoxin 1; LIAS—lipoic acid synthase; HSP70—heat shock protein 70; ERK—extracellular signal-regulated kinase; MAPK—mitogen-activated protein kinase; Cu^+^—cuprous ion; AChE—acetylcholinesterase; BDNF—brain-derived neurotrophic factor; SYN—synaptophysin; CREB—cAMP response element-binding protein; Camk2g—calcium/calmodulin-dependent protein kinase type II gamma chain; Adcyap1—adenylate cyclase activating polypeptide 1; Per1—period circadian regulator 1; Bax—Bcl-2-associated X protein; Bcl-2—B-cell lymphoma 2; NeuN—neuronal nuclei; iNOS—inducible nitric oxide synthase; nNOS—neuronal nitric oxide synthase; Ac-Tau—acetylated tau protein; Sirt1—sirtuin 1.

## Data Availability

No new data were created or analyzed in this study. Data sharing is not applicable to this article.
